# rAAV TGF-β and FGF-2 Overexpression via pNaSS-Grafted PCL Films Stimulates the Reparative Activities of Human ACL Fibroblasts

**DOI:** 10.3390/ijms241311140

**Published:** 2023-07-06

**Authors:** Mahnaz Amini, Jagadeesh K. Venkatesan, Tuan N. Nguyen, Wei Liu, Amélie Leroux, Henning Madry, Véronique Migonney, Magali Cucchiarini

**Affiliations:** 1 Center of Experimental Orthopaedics, Saarland University Medical Center, Kirrbergerstr. Bldg 37, 66421 Homburg, Germany; 2 LBPS/CSPBAT UMR CNRS 7244, Université Sorbonne Paris Nord, 93430 Villetaneuse, France

**Keywords:** human anterior cruciate ligament, gene transfer, rAAV, TGF-β, FGF-2, PCL, pNaSS grafting

## Abstract

Lesions in the human anterior cruciate ligament (ACL) are frequent, unsolved clinical issues due to the limited self-healing ability of the ACL and lack of treatments supporting full, durable ACL repair. Gene therapy guided through the use of biomaterials may steadily activate the processes of repair in sites of ACL injury. The goal of the present study was to test the hypothesis that functionalized poly(sodium styrene sulfonate)-grafted poly(ε-caprolactone) (pNaSS-grafted PCL) films can effectively deliver recombinant adeno-associated virus (rAAV) vectors as a means of overexpressing two reparative factors (transforming growth factor beta-TGF-β and basic fibroblast growth factor-FGF-2) in primary human ACL fibroblasts. Effective, durable rAAV reporter red fluorescent protein and candidate TGF-β and FGF-2 gene overexpression was achieved in the cells for at least 21 days, especially when pNaSS-grafted PCL films were used versus control conditions, such as ungrafted films and systems lacking vectors or films (between 1.8- and 5.2-fold differences), showing interactive regulation of growth factor production. The expression of TGF-β and FGF-2 from rAAV via PCL films safely enhanced extracellular matrix depositions of type-I/-III collagen, proteoglycans/decorin, and tenascin-C (between 1.4- and 4.5-fold differences) in the cells over time with increased levels of expression of the specific transcription factors Mohawk and scleraxis (between 1.7- and 3.7-fold differences) and without the activation of the inflammatory mediators IL-1β and TNF-α, most particularly with pNaSS-grafted PCL films relative to the controls. This work shows the value of combining rAAV gene therapy with functionalized PCL films to enhance ACL repair.

## 1. Introduction

Injuries in the anterior cruciate ligament (ACL), the ligament that provides essential knee stability, are highly prevalent in the human population, affecting more than 200,000 persons every year in the United States and with an incidence of 1/3000 in Europe only, with costs exceeding USD 7 billion per year [1,2], potentially resulting in osteoarthritis and disability [3,4].

The ACL is a dense, hierarchically organized tissue with a rich extracellular matrix (ECM) that is mostly composed of collagen fiber bundles comprised of type-I and also -III, -IV, -V, and -VI collagen and other structural components, such as proteoglycans/decorin, tenascin-C, fibronectin, elastin, and thrombospondin, that surround ECM-producing fibroblasts which are present at a hypocellular level in a cable-like structure [5]. As a result of its very low cell content, the ACL has a limited and slow intrinsic self-repair ability following injury that promotes the formation of scar tissue with poor mechanical strength [3]. While a variety of treatments are available to manage ACL lesions, including conservative regimens like immobilization and bracing, physiotherapy, and corticoid injection, and surgical options, including ACL reconstruction using auto-/allografts and synthetic materials and substitutes [1], thus far, none satisfactorily allow for complete, long-lasting, and safe repair of such injuries [6].

Gene therapy using the administration of therapeutic candidate sequences carried in a gene vector is a promising strategy to promote the effective and durable overexpression and biological (reparative) functions of therapeutic gene products in sites of ACL lesions relative to the use of recombinant factors with short pharmacological half-lives [7,8,9]. However, as a number of physiological barriers still preclude the effective application of gene therapy for translational purposes, including a potential dissemination of gene vectors to nontarget locations and their neutralization by host immune responses [9], significant efforts have been made in developing innovative systems based on the use of biocompatible materials that allow for the localized and safe overexpression of therapeutic gene sequences via a spatiotemporal delivery of the vehicles carrying them [9].

A variety of candidate genes have been applied to enhance ACL repair, among which growth factors, such as transforming growth factor beta (TGF-β) [10,11], basic fibroblast growth factor (FGF-2) [12], insulin-like growth factor I (IGF-I) [13], bone morphogenetic proteins (BMP-2, -6, -12, and -13) [14,15,16,17], platelet-derived growth factor (PDGF) [18], and vascular endothelial growth factor (VEGF) [11], are capable of stimulating healing activities (cell migration, proliferation, and adhesion, matrix deposition, angiogenesis, and mechanical stiffness). Transcription factors have also been applied in the form of candidate gene sequences, such as scleraxis [19,20] and Mohawk [21], to enhance cell differentiation and matrix deposition. While nonviral [17,18,20] and classical viral (adenoviral and retro-/lentiviral) vectors [10,11,13,14,15,16,19,21] have been generally employed in these studies, vehicles based on the human non-pathogenic adeno-associated virus (AAV) might provide improved gene shuttles for ACL repair. Recombinant AAV (rAAV) vectors are more potent than (i) nonviral vectors that are only expressed at low and transient levels, (ii) adenoviral vectors that also support very short-term transgene expression and are highly immunogenic, and (iii) retro-/lentiviral vectors that only target dividing cells [7,8,9]. Instead, rAAV vectors can modify nondividing cells, including ACL fibroblasts, at very high and permanent gene transfer efficiencies of up to 100% for months to years in a safer manner [12,22,23].

Yet the clinical use of rAAV remains challenging due to existing barriers in the joint that may impede the effective overexpression of the therapeutic sequences delivered, including the presence of neutralizing antibodies against the viral AAV capsid in a majority of individuals and patients [24]. Based on the innovative concept of spatiotemporal biomaterial-guided gene therapy [9], the goal of the present study was to examine the potential of functionalized poly(sodium styrene sulfonate)-grafted poly(ε-caprolactone) (pNaSS-grafted PCL) films [25,26,27] to deliver functional therapeutic rAAV vectors coding for the reparative TGF-β and FGF-2 factors [10,11,12] in primary human ACL fibroblasts as an innovative, biomaterial-guided gene therapy strategy [28,29] and future off-the-shelf tool for enhancing the processes of ACL repair in patients.

## 2. Results

### 2.1. Effective, Sustained rAAV-Mediated Gene Expression in hACL Fibroblasts via PCL Film-Guided Vector Delivery

**Figure 1:**
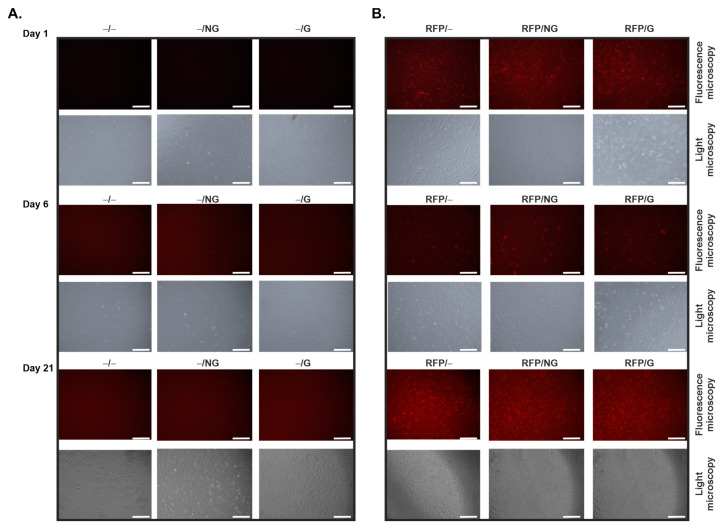
Detection of transgene (RFP) expression in hACL fibroblast cultures transduced using rAAV-RFP-coated PCL films. The PCL films were coated with rAAV-RFP (40 μL; 8 × 10^5^ transgene copies) or left without a vector coating before they were applied to the cultures, and RFP expression was examined under fluorescence microscopy at the denoted time points, as described in the Materials and Methods section ((**A**): absence of vector; (**B**): rAAV-RFP gene delivery; top panels: fluorescent photographs; lower panels: corresponding light microscopy photographs; magnification ×10; scale bars: 300 μm; all representative data). Abbreviations: −/−—lack of vector and PCL film; −/NG—uncoated, ungrafted PCL films; −/G—uncoated, pNaSS-grafted PCL films; RFP/−—film-free rAAV-RFP; RFP/NG—rAAV-RFP-coated, ungrafted PCL films; RFP/G—rAAV-RFP-coated, pNaSS-grafted PCL films.

Monolayer cultures of primary human ACL (hACL) fibroblasts were first transduced using the reporter rAAV-RFP vector coated on pNaSS-grafted versus ungrafted PCL films to examine the potential of this biomaterial-assisted gene transfer method to genetically modify these cells over time in vitro relative to control conditions in the absence of vector coating or to film-free rAAV gene transfer. The data reveal the successful, sustained expression of RFP via rAAV from day 1 until at least day 21 (the longest time-point evaluated) regardless of the type of PCL film employed, with signals similar to those achieved when using a film-free rAAV-RFP solution (Figure 1B) versus conditions in which rAAV-RFP was omitted (Figure 1A).

**Figure 2:**
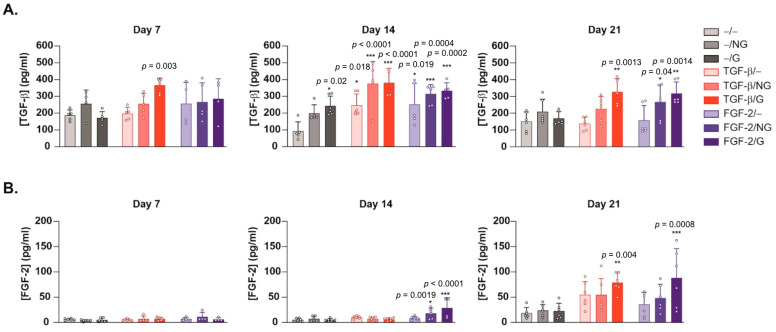
Detection of transgene (TGF-β, FGF-2) expression in hACL fibroblast cultures transduced using rAAV-coated PCL films via an ELISA. The PCL films were coated with rAAV-hTGF-β or rAAV-hFGF-2 (40 μL each vector; 8 × 10^5^ transgene copies) or left without a vector coating before they were applied to the cultures, and the expression levels of (**A**) TGF-β and (**B**) FGF-2 were examined at the denoted time points via a specific ELISA, as described in the Materials and Methods section. Abbreviations: −/−—lack of vector and PCL film; −/NG—uncoated, ungrafted PCL films; −/G—uncoated, pNaSS-grafted PCL films; TGF-β/−—film-free rAAV-hTGF-β; TGF-β/NG—rAAV-hTGF-β-coated, ungrafted PCL films; TGF-β/G—rAAV-hTGF-β-coated, pNaSS-grafted PCL films; FGF-2/−—film-free rAAV-hFGF-2; FGF-2/NG—rAAV-hFGF-2-coated, ungrafted PCL films; FGF-2/G—rAAV-hFGF-2-coated, pNaSS-grafted PCL films. Statistically significant versus the −/− condition (* *p* ≤  0.05, ** *p* ≤ 0.01, and *** *p* ≤ 0.001) (dots represent single replicates).

Monolayer cultures of primary hACL fibroblasts were then transduced using the candidate rAAV-hTGF-β and rAAV-hFGF-2 vectors coated on pNaSS-grafted versus ungrafted PCL films to monitor whether such a system was also capable of mediating the overexpression of TGF-β and FGF-2 in these cells over time in vitro relative to control conditions in the absence of vector coating. An estimation of the levels of TGF-β production via an ELISA showed a significant, optimal increase in growth factor synthesis in hACL fibroblasts already after 7 days when applying rAAV-hTGF-β-coated, pNaSS-grafted PCL films (TGF-β/G) relative to control conditions without a vector coating (−/−, −/G, −/NG) (up to a 2-fold difference, *p* ≤ 0.003) (Figure 2A). Significant TGF-β overexpression was sustained and still optimally noted after 14 and 21 days when using TGF-β/G relative to control conditions without a vector coating (up to 4- and 2.3-fold differences, *p* ≤ 0.001 and *p* ≤ 0.013, respectively) (Figure 2A). This was also observed, but only after 14 days, when applying rAAV-hTGF-β-coated, ungrafted PCL films (TGF-β/NG) or when using a film-free rAAV-hTGF-β solution (TGF-β/−) relative to control conditions without a vector coating (up to 4- and 2.4-fold differences, *p* ≤ 0.001 and *p* ≤ 0.018, respectively) (Figure 2A). Interestingly, the administration of rAAV-hFGF-2 via pNaSS-grafted PCL films (FGF-2/G) also significantly and optimally increased TGF-β synthesis in hACL fibroblasts after 14 and 21 days relative to control conditions without a vector coating (up to 3.6- and 2.2-fold differences, *p* ≤ 0.0002 and *p* ≤ 0.0014, respectively) (Figure 2A). This was also observed when applying rAAV-hFGF-2-coated, ungrafted PCL films (FGF-2/NG) after 14 and 21 days relative to control conditions without a vector coating (up to 3.1- and 1.8-fold differences, *p* ≤ 0.;004 and *p* ≤ 0.040, respectively) or when using a film-free rAAV-hFGF-2 solution after 14 days relative to control conditions without a vector coating (up to a 2.5-fold difference, *p* ≤ 0.019) (Figure 2A). An estimation of the levels of FGF-2 production via an ELISA showed a significant, optimal increase in growth factor synthesis in hACL fibroblasts after 14 days when applying rAAV-hFGF-2-coated, pNaSS-grafted PCL films (FGF-2/G) relative to control conditions without a vector coating (up to a 5.2-fold difference, *p* ≤ 0.001) (Figure 2B). Significant FGF-2 overexpression was sustained and still optimally noted after 21 days using FGF-2/G relative to control conditions without a vector coating (up to a 4.7-fold difference, *p* ≤ 0.0008) (Figure 2B). This was also observed but only after 14 days when applying rAAV-hFGF-2-coated, ungrafted PCL films (FGF-2/NG) relative to control conditions without a vector coating (up to a 3.3-fold difference, *p* ≤ 0.019) (Figure 2B). Interestingly, the administration of rAAV-hTGF-β via pNaSS-grafted PCL films (TGF-β/G) also significantly and optimally increased FGF-2 synthesis in hACL fibroblasts after 21 days relative to control conditions without a vector coating (up to a 4.3-fold difference, *p* ≤ 0.004) (Figure 2B).

**Figure 3:**
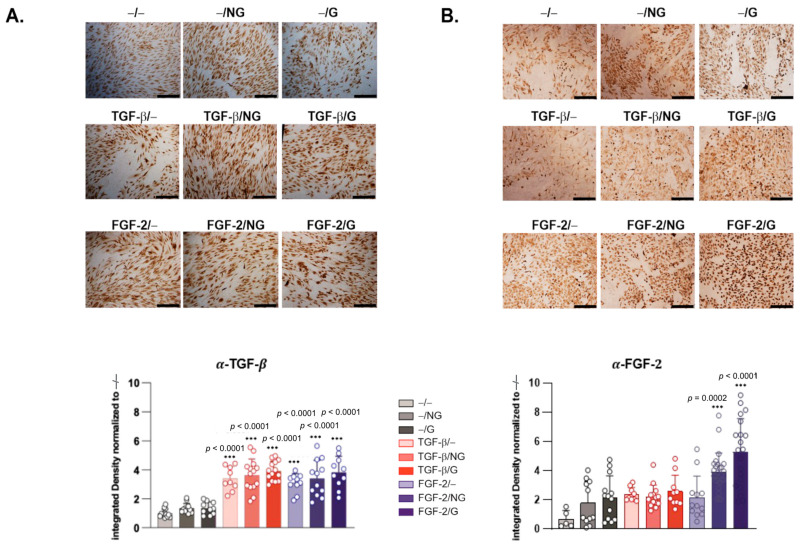
Detection of transgene (TGF-β, FGF-2) expression via immunocytochemistry in hACL fibroblast cultures transduced using rAAV-coated PCL films. The PCL films were coated with rAAV-hTGF-β or rAAV-hFGF-2 or left without a vector coating before they were applied to the cultures as described in Figure 2, and the expression of (**A**) TGF-β and (**B**) FGF-2 was examined after 21 days via immunocytochemistry (magnification ×4; scale bars: 300 μm; all representative data) with an analysis of the integrated densities using ImageJ software, as described in the Materials and Methods section. Abbreviations: −/−—lack of vector and PCL film; −/NG—uncoated, ungrafted PCL films; −/G—uncoated, pNaSS-grafted PCL films; TGF-β/−—film-free rAAV-hTGF-β; TGF-β/NG—rAAV-hTGF-β-coated, ungrafted PCL films; TGF-β/G—rAAV-hTGF-β-coated, pNaSS-grafted PCL films; FGF-2/−—film-free rAAV-hFGF-2; FGF-2/NG—rAAV-hFGF-2-coated, ungrafted PCL films; FGF-2/G—rAAV-hFGF-2-coated, pNaSS-grafted PCL films. Statistically significant versus the −/− condition (*** *p* ≤ 0.001) (dots represent single replicates).

Overall, these findings were corroborated via the immunocytochemical detection of transgene expression in hACL fibroblasts after 21 days with corresponding histomorphometric analyses (Figure 3), showing optimal expression of TGF-β (Figure 3A) and FGF-2 (Figure 3B) when applying rAAV-hTGF-β- and rAAV-hFGF-2-coated, pNaSS-grafted PCL films (TGF-β/G and FGF-2/G, respectively) relative to control conditions without a vector coating (up to 4.2- and 5.6-fold differences, respectively, *p* ≤ 0.0001). This was also observed when using rAAV-hTGF-β- and rAAV-hFGF-2-coated, ungrafted PCL films (TGF-β/NG and FGF-2/NG, respectively) relative to control conditions without a vector coating (up to 3.9- and 5-fold differences, *p* ≤ 0.0001 and *p* ≤ 0.0002, respectively) and with a film-free rAAV-hTGF-β solution (TGF-β/−) (up to a 3.7-fold difference, *p* ≤ 0.0001) (Figure 3A,B). Interestingly, the administration of rAAV-hFGF-2 via pNaSS-grafted PCL films (FGF-2/G) also significantly and optimally increased TGF-β synthesis in hACL fibroblasts after 21 days relative to control conditions without a vector coating (up to a 4.1-fold difference, *p* ≤ 0.0001) (Figure 3A). This was also observed when applying rAAV-hFGF-2-coated, ungrafted PCL films (FGF-2/NG) or when using a film-free rAAV-hFGF-2 solution (FGF-2/−) relative to control conditions without a vector coating (up to 3.7- and 3.2-fold differences, respectively, *p* ≤ 0.0001) (Figure 3A). Interestingly, the administration of rAAV-hTGF-β via pNaSS-grafted PCL films (TGF-β/G) also optimally increased FGF-2 synthesis in hACL fibroblasts after 21 days relative to control conditions without a vector coating (up to a 3-fold difference), although statistical significance was not reached (*p* ≥ 0.05), as also noted when applying rAAV-hTGF-β-coated, ungrafted PCL films (TGF-β/NG) or when using a film-free rAAV-hTGF-β solution (TGF-β/−) relative to control conditions without a vector coating (up to 2.8- and 2.9-fold differences, respectively, *p* ≥ 0.05) (Figure 3B).

### 2.2. Effects of rAAV-Mediated TGF-β and FGF-2 Overexpression via PCL Film-Guided Vector Delivery on the Biological Activities of hACL Fibroblasts

Monolayer cultures of primary hACL fibroblasts were finally transduced using the candidate rAAV-hTGF-β and rAAV-hFGF-2 vectors coated on pNaSS-grafted versus ungrafted PCL films to evaluate the effects of TGF-β and FGF-2 overexpression on the biological activities of these cells over time in vitro relative to control conditions in the absence of a vector coating.

**Figure 4:**
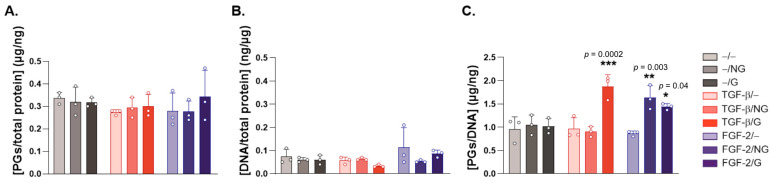
Evaluation of the proteoglycan and DNA contents in hACL fibroblast cultures transduced using rAAV-coated PCL films. The PCL films were coated with rAAV-hTGF-β or rAAV-hFGF-2 or left without a vector coating before they were applied to the cultures as described in Figure 2 and Figure 3, and the proteoglycan (PG) and DNA contents were monitored after 21 days (**A**) by assessing their binding to the DMMB dye and (**B**) via the Hoechst 33,258 assay, respectively, with normalization to the total protein contents and with (**C**) an estimation of the proteoglycan/DNA ratios, as described in the Materials and Methods section. Abbreviations: −/−—lack of vector and PCL film; −/NG—uncoated, ungrafted PCL films; −/G—uncoated, pNaSS-grafted PCL films; TGF-β/−—film-free rAAV-hTGF-β; TGF-β/NG—rAAV-hTGF-β-coated, ungrafted PCL films; TGF-β/G—rAAV-hTGF-β-coated, pNaSS-grafted PCL films; FGF-2/−—film-free rAAV-hFGF-2; FGF-2/NG—rAAV-hFGF-2-coated, ungrafted PCL films; FGF-2/G—rAAV-hFGF-2-coated, pNaSS-grafted PCL films. Statistically significant versus the −/− condition (* *p* ≤  0.05, ** *p* ≤ 0.01, and *** *p* ≤ 0.001) (dots represent single replicates).

While a direct estimation of the proteoglycan contents (Figure 4A) and DNA contents (Figure 4B) did not reveal significant effects of the candidate rAAV-hTGF-β- and rAAV-hFGF-2-coated ungrafted or pNaSS-grafted PCL films (TGF-β/NG, FGF-2/NG, TGF-β/G, and FGF-2/G) in hACL fibroblasts after 21 days relative to control conditions without a vector coating (−/−, −/G and −/NG) (*p* ≥ 0.05), there was a significant increase in the proteoglycan contents normalized to the DNA contents when applying the rAAV-hTGF-β-coated, pNaSS-grafted PCL films (TGF-β/G) and the rAAV-hFGF-2-coated ungrafted and pNaSS-grafted PCL films (FGF-2/NG and FGF-2/G) relative to control conditions without a vector coating (up to 1.8-, 1.6-, and 1.4-fold differences with TGF-β/G, FGF-2/NG, and FGF-2/G, *p* ≤ 0.0002, *p* ≤ 0.003, and *p* ≤ 0.040, respectively) (Figure 4C). An estimation of the cell viability indexes demonstrated the safety of the gene transfer approach regardless of the type of PCL film employed and the candidate vector applied (TGF-β/NG, FGF-2/NG, TGF-β/G, or FGF-2/G), as also noted in all other controls without a vector coating (−/G or −/NG) or with film-free rAAV solutions (TGF-β/− or FGF-2/−), with values ranging between 95% and 100% relative to the control (−/−) condition.

**Figure 5:**
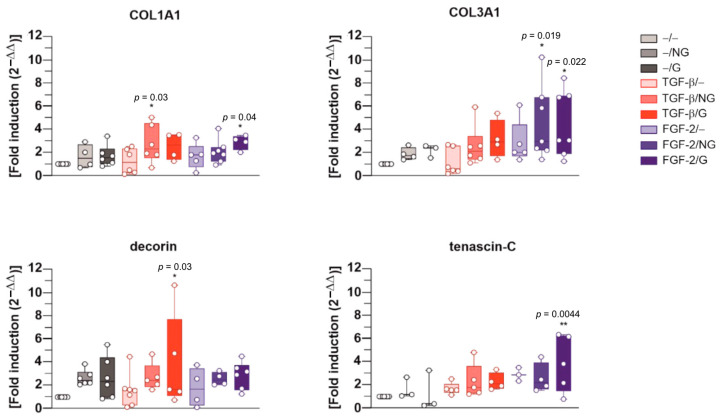
Evaluation of the gene expression profiles via real-time RT-PCR of the matrix components in hACL fibroblast cultures transduced using rAAV-coated PCL films. The PCL films were coated with rAAV-hTGF-β or rAAV-hFGF-2 or left without a vector coating before they were applied to the cultures as described in Figure 2, Figure 3 and Figure 4, and the gene expression profiles for type-I collagen (COL1A1), type-III collagen (COL3A1), decorin, and tenascin-C were measured after 21 days via real-time RT-PCR, with GAPDH serving as a housekeeping gene, as described in the Materials and Methods section. Ct values were obtained for each target gene and for GAPDH as a control for normalization, and fold inductions (relative to the −/− condition) were measured using the 2^−ΔΔCt^ method. Abbreviations: −/−—lack of vector and PCL film; −/NG—uncoated, ungrafted PCL films; −/G—uncoated, pNaSS-grafted PCL films; TGF-β/−—film-free rAAV-hTGF-β; TGF-β/NG—rAAV-hTGF-β-coated, ungrafted PCL films; TGF-β/G—rAAV-hTGF-β-coated, pNaSS-grafted PCL films; FGF-2/−—film-free rAAV-hFGF-2; FGF-2/NG—rAAV-hFGF-2-coated, ungrafted PCL films; FGF-2/G—rAAV-hFGF-2-coated, pNaSS-grafted PCL films. Statistically significant versus the −/− condition (* *p* ≤  0.05, ** *p* ≤ 0.01) (dots represent single replicates).

An evaluation of the gene expression profiles for matrix components via a real-time RT-PCR revealed significant increases in COL1A1 expression in hACL fibroblasts after 21 days when applying rAAV-hTGF-β-coated, ungrafted PCL films (TGF-β/NG) and rAAV-hFGF-2-coated, pNaSS-grafted PCL films (FGF-2/G) relative to control conditions without a vector coating (up to a 2.8-fold difference, *p* ≤ 0.030 and *p* ≤ 0.040, respectively), with a trend toward increased COL1A1 expression with rAAV-hTGF-β-coated, pNaSS-grafted PCL films (TGF-β/G) (2.5-fold difference, *p* ≥ 0.050) or when using film-free rAAV-hTGF-β and rAAV-hFGF-2 solutions (TGF-β/− and FGF-2/−, respectively) (up to 2.4- and 2.3-fold differences, respectively, *p* ≥ 0.050) (Figure 5). Increased COL3A1 expression was also significantly noted in hACL fibroblasts after 21 days when applying rAAV-hFGF-2-coated ungrafted and pNaSS-grafted PCL films (FGF-2/N and, FGF-2G) relative to control conditions without a vector coating (up to a 4.5-fold difference, *p* ≤ 0.022), with a trend toward increased COL3A1 with rAAV-hTGF-β-coated ungrafted and pNaSS-grafted PCL films (TGF-β/NG and TGF-β/G) (up to 2.5- and 3.1-fold differences, respectively, *p* ≥ 0.050) or when using film-free rAAV-hTGF-β and rAAV-hFGF-2 solutions (TGF-β/− and FGF-2/−, respectively) (up to 2.2- and 4.1-fold differences, respectively, *p* ≥ 0.050) (Figure 5). There was also a significant increase in decorin expression in hACL fibroblasts after 21 days when applying rAAV-hTGF-β-coated pNaSS-grafted PCL films (TGF-β/G) relative to control conditions without a vector coating (up to a 3.8-fold difference, *p* ≤ 0.030), with a trend toward increased decorin expression with rAAV-hTGF-β-coated, ungrafted PCL films (TGF-β/NG) and rAAV-hFGF-2-coated, pNaSS-grafted PCL films (FGF-2/G) (up to 2.7- and 2.8-fold differences, respectively, *p* ≥ 0.050) or when using film-free rAAV-hTGF-β and rAAV-hFGF-2 solutions (TGF-β/− and FGF-2/−, respectively) (up to 1.4- and 3.1-fold differences, respectively, *p* ≥ 0.050) (Figure 5). Finally, a significant increase in tenascin-C was observed in hACL fibroblasts after 21 days when applying rAAV-hFGF-2-coated, pNaSS-grafted PCL films (FGF-2/G) relative to control conditions without a vector coating (up to a 3.8-fold difference, *p* ≤ 0.004), with a trend toward increased tenascin-C expression with rAAV-hTGF-β-coated, pNaSS-grafted PCL films (TGF-β/G) and rAAV-hFGF-2-coated, ungrafted PCL films (FGF-2/NG) (up to 2.3- and 2.6-fold differences, *p* ≥ 0.050) or when using film-free rAAV-hTGF-β and rAAV-hFGF-2 solutions (TGF-β/− and FGF-2/−, respectively) (up to 2.1- and 3-fold differences, respectively, *p* ≥ 0.050) (Figure 5).

**Figure 6:**
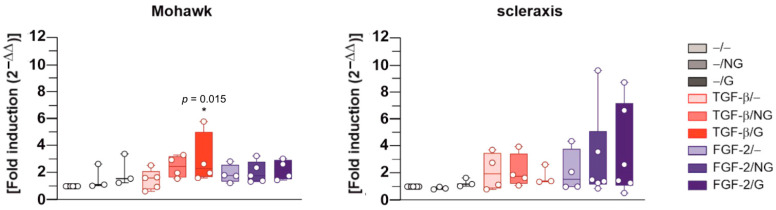
Gene expression profiles of transcription factors in hACL fibroblast cultures transduced with rAAV-coated PCL films, evaluated via real-time RT-PCR. The PCL films were coated with rAAV-hTGF-β or rAAV-hFGF-2 or left without a vector coating before they were applied to the cultures as described in Figure 2, Figure 3, Figure 4 and Figure 5, and the gene expression profiles for Mohawk and scleraxis were measured after 21 days via real-time RT-PCR with GAPDH serving as a housekeeping gene, as described in the Materials and Methods section. Ct values were obtained for each target gene and for GAPDH as a control for normalization, and fold inductions (relative to the −/− condition) were measured using the 2^−ΔΔCt^ method. Abbreviations: −/−—lack of vector and PCL film; −/NG—uncoated, ungrafted PCL films; −/G—uncoated, pNaSS-grafted PCL films; TGF-β/−—film-free rAAV-hTGF-β; TGF-β/NG—rAAV-hTGF-β-coated, ungrafted PCL films; TGF-β/G—rAAV-hTGF-β-coated, pNaSS-grafted PCL films; FGF-2/−—film-free rAAV-hFGF-2; FGF-2/NG—rAAV-hFGF-2-coated, ungrafted PCL films; FGF-2/G—rAAV-hFGF-2-coated, pNaSS-grafted PCL films. Statistically significant versus the −/− condition (* *p* ≤ 0.05) (dots represent single replicates).

Interestingly, an evaluation of the gene expression profiles for specific transcription factors via real-time RT-PCR revealed significant increases in Mohawk expression in hACL fibroblasts after 21 days when applying rAAV-hTGF-β-coated, pNaSS-grafted PCL (TGF-β/G) relative to control conditions without a vector coating (up to a 3-fold difference, *p* ≤ 0.015), with a trend toward increased Mohawk expression with rAAV-hTGF-β-coated, ungrafted PCL films (TGF-β/NG) and rAAV-hFGF-2-coated ungrafted and pNaSS-grafted PCL films (FGF-2/NG, FGF-2/G) (up to 2.4-, 2-, and 2.2-fold differences, *p* ≥ 0.050) or when using film-free rAAV-hTGF-β and rAAV-hFGF-2 solutions (TGF-β/− and FGF-2/−, respectively) (up to 2- and 2.5-fold differences, respectively, *p* ≥ 0.050) (Figure 6). There was also a trend toward increased scleraxis expression when applying rAAV-hTGF-β- and rAAV-hFGF-2-coated ungrafted and pNaSS-grafted PCL films (TGF-β/NG, TGF-β/G, FGF-2/NG, and FGF-2/G) relative to control conditions without a vector coating (up to 2.1-, 1.7-, 3-, and 3.5-fold differences, *p* ≥ 0.050) or when using film-free rAAV-hTGF-β and rAAV-hFGF-2 solutions (TGF-β/− and FGF-2/−, respectively) (up to 3.6- and 3.7-fold differences, respectively, *p* ≥ 0.050) (Figure 6).

**Figure 7:**
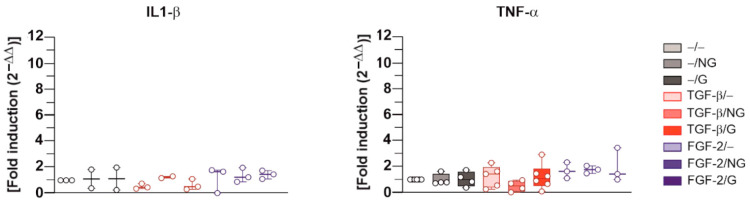
Gene expression profiles of inflammatory mediators in hACL fibroblast cultures transduced with rAAV-coated PCL films, evaluated via real-time RT-PCR. The PCL films were coated with rAAV-hTGF-β or rAAV-hFGF-2 or left without a vector coating before they were applied to the cultures as described in Figure 2, Figure 3, Figure 4, Figure 5 and Figure 6, and the gene expression profiles for IL-1β and TNF-α were measured after 21 days via real-time RT-PCR with GAPDH serving as a housekeeping gene, as described in the Materials and Methods section. Ct values were obtained for each target gene and for GAPDH as a control for normalization, and fold inductions (relative to the −/− condition) were measured using the 2^−ΔΔCt^ method. Abbreviations: −/−—lack of vector and PCL film; −/NG—uncoated, ungrafted PCL films; −/G—uncoated, pNaSS-grafted PCL films; TGF-β/−—film-free rAAV-hTGF-β; TGF-β/NG—rAAV-hTGF-β-coated, ungrafted PCL films; TGF-β/G—rAAV-hTGF-β-coated, pNaSS-grafted PCL films; FGF-2/−—film-free rAAV-hFGF-2; FGF-2/NG—rAAV-hFGF-2-coated, ungrafted PCL films; FGF-2/G—rAAV-hFGF-2-coated, pNaSS-grafted PCL films (dots represent single replicates).

Finally, and of further critical importance, the application of the rAAV-hTGF-β- or rAAV-hFGF-2-coated ungrafted and pNaSS-grafted PCL films (TGF-β/NG, TGF-β/G, FGF-2/NG, and FGF-2/G) did not trigger the expression of pro-inflammatory IL-1β or TNF-α mediators in the hACL fibroblasts after 21 days relative to control conditions without a vector coating (*p* ≥ 0.050) (Figure 7).

## 3. Discussion

The combination of gene therapy and the use of biocompatible materials is a promising tool for improving the processes of ACL repair upon the delivery of therapeutic gene sequences in a safe and controlled spatiotemporal manner that allows for future non-invasive translation in patients [9]. Among a variety of candidate factors, TGF-β and FGF-2 were selected here in light of their reparative properties [10,11,12] and tested for their competence in activating hACL fibroblasts upon their delivery via clinically adapted rAAV gene transfer vectors guided by the application of functionalized (pNaSS-grafted) PCL films [28,29].

The data first reveal that the PCL films were capable of successfully delivering and expressing reporter (RFP) rAAV vectors in hACL fibroblasts over extended periods of time (at least 21 days, the longest time-point examined), as noted with a film-free vector solution versus control conditions without a vector, a result which is in good agreement with the findings achieved when applying the current rAAV-RFP construct via similar PCL films to human bone marrow aspirates at a similar MOI [28], probably due to the effective release of a vector coating from such a biomaterial [28]. The data next demonstrate that the PCL films were also capable of successfully delivering and expressing the two candidate (TGF-β and FGF-2) rAAV vectors in hACL fibroblasts over extended periods of time (at least 21 days, the longest time-point examined), especially when applying pNaSS-grafted PCL films versus control conditions and when compared with film-free vector solutions, results that are in good agreement with previous findings achieved when applying the current rAAV-hTGF-β construct via similar PCL films to human bone marrow aspirates at a similar MOI [29], which again reflects the effectiveness of the vector coating released over time from this biomaterial [28] and extends our work using the current rAAV-hFGF-2 construct applied as a vector solution to hACL fibroblasts at a similar MOI [12]. Remarkably, an interactive regulation of growth factor production was noted here in hACL fibroblasts in concordance with previous work showing TGF-β/FGF-2 synergistic interactions in articular chondrocytes [30].

The results next show that the successful, durable overexpression of the two candidate (TGF-β, FGF-2) rAAV vectors enhanced the deposition of typical ECM compounds (type-I and -III collagen, proteoglycans/decorin, and tenascin-C) in hACL fibroblasts over extended periods of time (at least 21 days, the longest time-point examined) in a safe manner (95–100% cell viability), especially when applying pNaSS-grafted PCL films versus control conditions and when compared with film-free vector solutions, results which are in good agreement with our previous findings using the current rAAV-hTGF-β construct delivered via similar PCL films to human bone marrow aspirates [29] and extending our work using the current rAAV-hFGF-2 construct applied as a vector solution to hACL fibroblasts [12], which is overall concordant with the properties of the growth factors, especially the TGF-β1 isoform applied in this study [10,31,32,33,34]. These effects were accompanied by and probably due to increased levels of specific ECM-inducing transcription factors, Mohawk and scleraxis [20,35], in the hACL fibroblasts over extended periods of time (at least 21 days), especially when applying pNaSS-grafted PCL films versus control conditions and when compared with film-free vector solutions, results which are in overall agreement with the properties of these growth factors [36,37] and which extend our work using the current rAAV-hFGF-2 construct applied as a vector solution to hACL fibroblasts [12]. It is equally important that the delivery of rAAV-hTGF-β and rAAV-hFGF-2 via the PCL films had no detrimental effects on inflammatory processes (the production of IL-1β and TNF-α) in the hACL fibroblasts over time (at least 21 days), in concordance with the properties of these growth factors [38,39,40] and with the protective effects of the PCL films, especially following pNaSS grafting [26].

The present study shows the potential value of delivering clinically suited rAAV vectors that code for TGF-β and FGF-2 via pNaSS-grafted PCL films as an off-the-shelf novel system for activating the processes of ACL repair. It remains to be seen whether a concomitant application of both factors via such a material might even be more beneficial to achieving this goal as a combination of rAAV vectors can be conveniently envisaged without interference [41]. There is ongoing work to test the current approach as a therapeutic platform in relevant animal models of ACL lesions in vivo [42] that might be more potent, safe, and durable than the use of recombinant factors to heal sites of ACL injury [43,44].

## 4. Materials and Methods

### 4.1. Reagents

All reagents were purchased from Sigma-Aldrich (Munich, Germany), including 4-styrenesulfonic acid sodium salt hydrate (NaSS) (cat. no. 434574), unless otherwise indicated. The anti-TGF-β (V) antibody was obtained from Invitrogen (ThermoFisher Scientific, Karlsruhe, Germany), and the anti-FGF-2 (C-18) antibody was obtained from Santa Cruz Biotechnology (Heidelberg, Germany). The biotinylated secondary antibodies and the ABC reagent were obtained from Vector Laboratories (Alexis Deutschland GmbH, Grünberg, Germany). The AAVanced Concentration Reagent was purchased at System Bioscience (Heidelberg, Germany). The TGF-β Quantikine Enzyme-Linked Immunosorbent Assay (ELISA; DB100B) and the FGF-2 Quantikine ELISA (DFB50) were obtained from R&D Systems (Mannheim, Germany). The Cell Proliferation Reagent WST-1 was obtained from Roche Applied Science (Mannheim, Germany).

### 4.2. Isolation and Culture of Primary Human Anterior Cruciate Ligament (hACL) Fibroblasts

**Figure 8:**
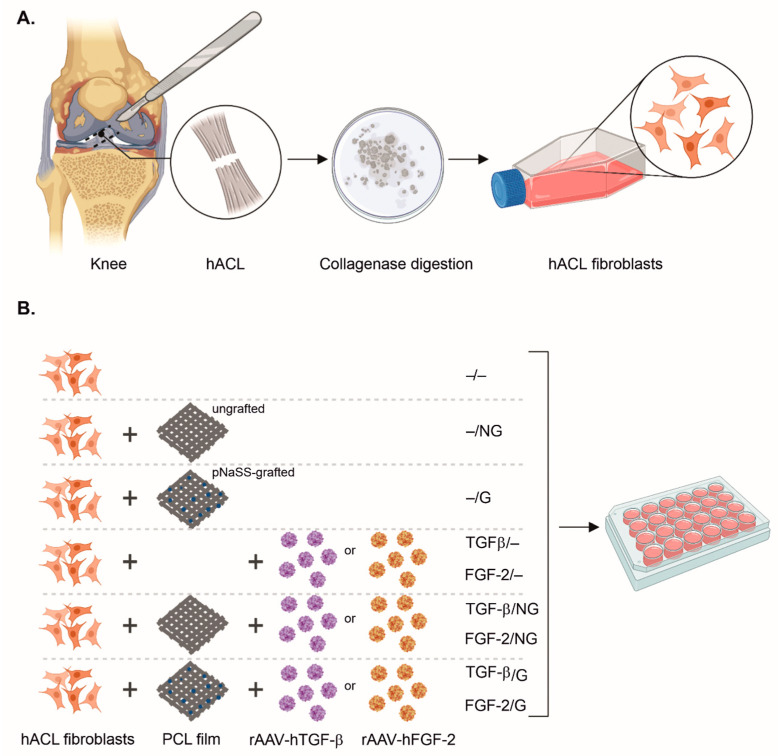
Study design. (**A**) Preparation of hACL fibroblasts. (**B**) Experimental gene transfer conditions in hACL fibroblasts (10^4^ cells; 40 μL rAAV vectors, i.e., 8 × 10^5^ transgene copies per PCL film or as film-free solutions, MOI = 80). Abbreviations: −/−—lack of vector and PCL film; −/NG—uncoated, ungrafted PCL films; −/G—uncoated, pNaSS-grafted PCL films; TGF-β/−—film-free rAAV-hTGF-β; FGF-2/−—film-free rAAV-hFGF-2; TGF-β/NG—rAAV-hTGF-β-coated, ungrafted PCL films; FGF-2/NG—rAAV-hFGF-2-coated, ungrafted PCL films; TGF-β/G—rAAV-hTGF-β-coated, pNaSS-grafted PCL films; FGF-2/G—rAAV-hFGF-2-coated, pNaSS-grafted PCL films.

Human anterior cruciate ligament (hACL) fibroblasts were obtained from donors undergoing total knee arthroplasty (*n* = 3, age range 72–78 years) [12]. Only ligaments without tears or visible degenerative changes upon gross examination were used. The study was approved by the Ethics Committee of the Saarland Physicians Council (Ärztekammer des Saarlandes, reference number Bu291/20). All patients provided informed consent before their inclusion in the evaluation, which was performed according to the Helsinki Declaration. After the retrieval of ACL tissue, small tissue pieces (1–2 mm^2^) were incubated in 0.2% (*w*/*v*) collagenase in Dulbecco’s Modified Eagle’s Medium (DMEM), 2% penicillin–streptomycin (pen-strep) for 24 h at 37 °C under 5% CO_2_ [12] (Figure 8A). The suspension was then centrifuged 5 min at 1500 rpm, and the clot was first resuspended in DMEM for centrifugation (5 min, 1500 rpm) and then in DMEM complemented with 10% fetal bovine serum (FBS) and 1% pen-strep [12]. The cells were maintained in T-75 flasks at 37 °C under 5% CO_2_ until confluence was reached (Figure 8A). The cells were then seeded at a density of 10^4^ cells/well in a 24-well plate and maintained at 37 °C under 5% CO_2_ for up to 21 days [12].

### 4.3. rAAV Vectors

rAAV vectors were created using a parental AAV-2 genomic clone (pSSV9) [45,46]. The rAAV-RFP vector carries the Discosoma sp. red fluorescent protein (RFP) gene, the rAAV-hTGF-β vector carries a 1.2 kb human transforming growth factor beta 1 (hTGF-β) cDNA sequence, and the rAAV-hFGF-2 vector carries a human basic fibroblast growth factor (hFGF-2) cDNA sequence; all are controlled by the cytomegalovirus immediate-early (CMV-IE) promoter [12,28,29,47,48]. Helper-free (two-plasmid) transfection in HEK 293 cells was used to package conventional vectors (not self-complementary), employing the packaging plasmid pXX2 and the adenovirus helper plasmid pXX6 [28,29,48]. The vectors were purified using AAVanced Concentration Reagent [28]. The vector preparations were titered via real-time PCR [12,28,29,47,48], reaching ~10^10^ transgene copies/mL (i.e., ~1/500 functional recombinant viral particles).

### 4.4. Poly(ε-Caprolactone) Films

The poly(ε-caprolactone) (PCL) films (Figure 8B) were created via spin coating using an SPIN150-v3 SPS [25,26,27]. PCL (60% [*w*/*v*] in dichloromethane) was dropped for spinning on a glass slide (30 s at 1500 rpm). The films were air-dried for 2 h, followed by vacuum drying for 24 h. The films were then cut into 4 mm disks, and some films were grafted with poly(sodium styrene sulfonate) (pNaSS) (1.3 × 10^−5^ mol/g) via ozonation (10 min at 30 °C), followed by graft polymerization (3 h at 45 °C) in degassed NaSS (15% *w*/*v* in distilled water) (Figure 8B). The films were rinsed and vacuum-dried after they were washed in distilled water, 0.15 M of NaCl, and phosphate-buffered saline.

### 4.5. rAAV Immobilization on PCL Films

The PCL films were sterilized with 70% ethanol (10 min incubation) and washed with PBS prior to incubation with 0.002% poly-L-lysine overnight at 37 °C [28,29]. After the films were washed with PBS twice, the rAAV vectors (40 μL, 8 × 10^5^ transgene copies, MOI = 80) were immobilized on the films for 2 h via dropping at 37 °C (rAAV-coated PCL films) [28,29] (Figure 8B). Some PCL films were prepared without the addition of rAAV vectors as controls (Figure 8B). Controlled release studies were not performed here as we previously reported that all PCL films (grafted and ungrafted) employed herein effectively release rAAV over extended periods of time (at least 21 days) [28].

### 4.6. rAAV-Mediated Gene Transfer

rAAV-coated or uncoated (pNaSS-grafted and ungrafted) PCL films were placed on the bottoms of 24-well plates seeded with hACL fibroblasts (10^4^ cells/well) and incubated for 2 h at 37 °C under 5% CO_2_. They were then incubated with DMEM supplemented with 10% FBS, and 1% pen-strep was added for an overnight incubation at 37 °C under 5% CO_2_ (Figure 8B). Some wells were used as control conditions by directly adding film-free rAAV vector solutions to the cells or left without rAAV vectors (Figure 8B). The medium was replaced after 24 h and every two days for up to 21 days. When provided, the PCL films were left over the entire period of prolonged culture.

### 4.7. Detection of Transgene Expression

RFP expression was monitored under fluorescence microscopy using a 568 nm filter (Olympus CK41; Olympus, Hamburg, Germany) [28,47]. The expression of TGF-β and FGF-2 was monitored at the denoted time points using specific ELISAs according to the manufacturer’s instructions [12,29,47,48]. A GENios spectrophotometer/fluorometer (Tecan, Crailsheim, Germany) was used for the measurements [12,29,47,48]. TGF-β and FGF-2 expression was also monitored by immunocytochemistry using specific primary antibodies, biotinylated secondary antibodies, and the ABC method with diaminobenzidine (DAB) as a chromogen for evaluation under light microscopy (Olympus BX45) [12,29,47,48]. To control for secondary immunoglobulins, samples were processed with the omission of the primary antibody.

### 4.8. Histomorphometric Analysis

A histomorphometric analysis of transgene (TGF-β, FGF-2) expression, monitored via immunocytochemistry, was performed via estimating the integrated densities, using ImageJ software (https://imagej.nih.gov/ij) (National Institutes of Health, Bethesda, MD, USA).

### 4.9. Biological Analyses

The cultures were collected and digested with papain as previously described [12,29,47,48]. Cell viability was assessed with Cell Proliferation Reagent WST-1, with optical densities (OD^450 nm^) being proportional to the cell numbers [29] and the % of cell viability determined as % viability = [OD^450 nm^ (test condition)/OD^450 nm^ (condition lacking both rAAV vector and PCL film, i.e., −/−)] × 100. The proteoglycan contents were assessed via binding to dimethyl methylene blue (DMMB) dye, the DNA contents were assessed via the Hoechst 33,258 assay, and the total protein contents were assessed via a Pierce BCA Protein assay for normalization (Pierce Thermo Scientific Protein Assay, Thermo Fisher Scientific) [12,29,47,48]. A GENios spectrophotometer/fluorometer (Tecan, Crailsheim, Germany) was used for the measurements [12,29,47,48].

### 4.10. Real-Time RT-PCR Analysis

The total cellular RNA of the cultures was extracted using an RNeasy Protect Mini Kit with an on-column RNase-free DNase treatment (Qiagen, Hilden, Germany) [28,29]. RNA was eluted in 30 μL RNase-free water, and reverse transcription was then performed using 8 μL of eluate with a 1st Strand cDNA Synthesis kit for RT-PCR (AMV) (Roche Applied Science) [28,29]. Real-time PCR amplification was tested using an Mx3000P QPCR system (Stratagene, Agilent Technologies, Waldbronn, Germany), using 3 μL of cDNA product with the Brilliant SYBR Green QPCR Master Mix (Stratagene Agilent Technologies) [28,29] and the following protocol: 10 min (95 °C), 55 amplification cycles (30 s denaturation at 95 °C; 1 min annealing at 55 °C; 30 s extension at 72 °C), denaturation 1 min (95 °C), and a final incubation 30 s (55 °C). The primers (150 nM final concentration; Invitrogen, ThermoFisher Scientific) used were (Table 1) type-I collagen (COL1A1, ligament marker), type-III collagen (COL3A1, ligament marker), decorin (leucine-rich proteoglycan), tenascin-C (ECM component), Mohawk (specific transcription factor), scleraxis (specific transcription factor), interleukin 1 beta (IL-1β, pro-inflammatory marker), tumor necrosis alpha (TNF-α, pro-inflammatory marker), and glyceraldehyde-3-phosphate dehydrogenase (GAPDH) as a housekeeping gene and internal control. The control reactions included water and non-reverse-transcribed mRNA, and the specificities of the products were confirmed via a melting curve analysis and agarose gel electrophoresis. The threshold cycle (Ct) value for each gene of interest was determined for each amplified sample by using MxPro QPCR software (https://www.agilent.com/en/product/real-time-pcr-%28qpcr%29/real-time-pcr-%28qpcr%29-instruments/mx3000-mx3005p-real-time-pcr-system-software/mxpro-qpcr-software-232751) (Stratagene Agilent Technologies), and the values were normalized to GAPDH expression via the 2^−ΔΔCt^ method [28,29].

### 4.11. Statistical Analysis

All experiments were repeated a minimum of three times (five times for the ELISA experiments) using all isolated hACL donor fibroblasts. A nonparametric one-way ANOVA was used for statistical analysis (except for biochemistry data, which were analyzed using Student’s *t*-test), with * *p* ≤  0.05, ** *p* ≤ 0.01, and *** *p* ≤ 0.001 considered statistically significant.

## Figures and Tables

**Table 1:** Primers employed in the study.

Gene	Forward Primer	Reverse Primer
COL1A1	5′-ACGTCCTGGTGAAG TTGGTC-3′	5′-ACCAGGGAAGCCTCTCTCTC-3′
COL3A1	5′-CACAAGGAGTCTGCATGTCT-3′	5′-GTTCACCAGGCTCACCAGCA-3′
decorin	5′-ACCCACTGAAGAGCTCAGGA-3′	5′-GCCATTGTCAACAGCAGAGA-3′
tenascin-C	5′-TCACATCCAGGTGCTTATTCC-3′	5′-CTAGAGTGTCTCACTATCAGG-3′
Mohawk	5′-AAGATACTCTTGGCGCTCGG-3′	5′-ACACTAAGCCGCTCAGCATT-3′
scleraxis	5′-TACCTGGGTTTTCTTCTGGTCACT-3′	5′-TATCAAAGACACAAGATGCCAGC-3′
IL-1β	5′- CCGTGCCTACGAACATGTC-3′	5′-CACACAGAAGCTCATCGGAG-3′
TNF-α	5′-AGAACCCCCTGGAGATAACC-3′	5′-AAGTGCAGCAGGCAGAAGAG-3′
GAPDH	5′-GAAGGTGAAGGTCGGAGTC-3′	5′-GAAGATGGTGATGGGATTTC-3′

## Data Availability

Data are available upon reasonable request.
